# Functional Consequences of Sulfhydryl Modification of the γ-Aminobutyric Acid Transporter 1 at a Single Solvent-Exposed Cysteine Residue

**DOI:** 10.1007/s00232-012-9492-9

**Published:** 2012-08-24

**Authors:** Jaison J. Omoto, Matthew J. Maestas, Ali Rahnama-Vaghef, Ye E. Choi, Gerardo Salto, Rachel V. Sanchez, Cynthia M. Anderson, Sepehr Eskandari

**Affiliations:** Biological Sciences Department, California State Polytechnic University, Pomona, 3801 West Temple Avenue, Pomona, CA 91768-4032 USA

**Keywords:** Neurotransmitter:sodium symporter, SLC6, GABA transporter, SLC6A1, Sulfhydryl modification, Cysteine 74

## Abstract

The aims of this study were to optimize the experimental conditions for labeling extracellularly oriented, solvent-exposed cysteine residues of γ-aminobutyric acid transporter 1 (GAT1) with the membrane-impermeant sulfhydryl reagent [2-(trimethylammonium)ethyl]methanethiosulfonate (MTSET) and to characterize the functional and pharmacological consequences of labeling on transporter steady-state and presteady-state kinetic properties. We expressed human GAT1 in *Xenopus laevis* oocytes and used radiotracer and electrophysiological methods to assay transporter function before and after sulfhydryl modification with MTSET. In the presence of NaCl, transporter exposure to MTSET (1–2.5 mM for 5–20 min) led to partial inhibition of GAT1-mediated transport, and this loss of function was completely reversed by the reducing reagent dithiothreitol. MTSET treatment had no functional effect on the mutant GAT1 C74A, whereas the membrane-permeant reagents *N*-ethylmaleimide and tetramethylrhodamine-6-maleimide inhibited GABA transport mediated by GAT1 C74A. Ion replacement experiments indicated that MTSET labeling of GAT1 could be driven to completion when valproate replaced chloride in the labeling buffer, suggesting that valproate induces a GAT1 conformation that significantly increases C74 accessibility to the extracellular fluid. Following partial inhibition by MTSET, there was a proportional reduction in both the presteady-state and steady-state macroscopic signals, and the functional and pharmacological properties of the remaining signals were indistinguishable from those of unlabeled GAT1. Therefore, covalent modification of GAT1 at C74 results in completely nonfunctional as well as electrically silent transporters.

## Introduction

In the nervous system, removal of the inhibitory neurotransmitter γ-aminobutyric acid (GABA) from the extracellular space of neurons and glia is accomplished by electrogenic Na^+^- and Cl^−^-coupled GABA transporters (GATs) (Borden 1996; Nelson 1998; Dalby 2003; Richerson and Wu 2003; Conti et al. 2004). Four GAT isoforms exist in mammalian tissues and belong to the large neurotransmitter/Na^+^ symporter family (NSS; 2.A.22 according to the transporter classification system; SLC6 according to the Human Genome Organization classification) (Nelson 1998; Busch and Saier 2002; Chen et al. 2004; Saier et al. 2006). Two sets of nomenclature systems are used to refer to the GATs: rat/human GAT-1, BGT-1, GAT-2 and GAT-3 correspond to mouse GAT1, GAT2, GAT3 and GAT4, respectively. GAT1 was the first family member to be cloned (Guastella et al. 1990; Nelson et al. 1990) and paved the way for the cloning and characterization of the related serotonin, dopamine, norepinephrine and glycine transporters (Blakely et al. 1991; Hoffman et al. 1991; Kilty et al. 1991; Pacholczyk et al. 1991; Shimada et al. 1991; Usdin et al. 1991; Guastella et al. 1992; Liu et al. 1992). GAT1 is the most abundant isoform within the nervous system and is expressed in both neurons and glia (Conti et al. 2004; Madsen et al. 2010). Tiagabine, a specific inhibitor of GAT1, is used clinically as an anticonvulsant drug and highlights the importance of GAT1 in maintaining GABA homeostasis in the brain extracellular fluid (Dalby 2003; Schousboe et al. 2011).

Chemical modification of endogenous or engineered cysteine residues has served as an important tool for structure–function studies of membrane proteins (Frillingos et al. 1998; Rudnick 2006; Guan and Kaback 2007; see also Anderson et al. 2010); and, in particular, when coupled with high-resolution information from crystal structures, sulfhydryl modification has served as a powerful tool to address specific questions regarding the functional role of targeted residues. Ideally, individual cysteine residues are engineered in the primary protein sequence against a cysteine-less background. Covalent modification of the introduced cysteine with thiol-specific probes, many of which differ in chemical properties, would then provide insight regarding the role that the residue in question plays in protein function. In practice, however, cysteine-less membrane proteins are difficult to study as they may not be properly trafficked to the plasma membrane or may be nonfunctional. Therefore, many investigators have relied on labeling endogenous cysteines in order to glean insight about protein function. This happens to be the case with GAT1. The extracellularly exposed, endogenous cysteine residue of GAT1, cysteine 74 (C74), has been used to gain insight into the nature and functional consequences of sulfhydryl modification at this residue (Bennett and Kanner 1997; Yu et al. 1998; Golovanevsky and Kanner 1999; Li et al. 2000; Kanner 2003; Zomot and Kanner 2003; Zhou et al. 2004, 2006; Zhou and Kanner 2005; Zomot et al. 2005; Mari et al. 2006; Rosenberg and Kanner 2008; Ben-Yona and Kanner 2009; Meinild et al. 2009). While it has been shown that sulfhydryl modification at C74 renders the transporter nonfunctional, the mechanism of this effect is not known. In previous studies, rather harsh labeling conditions were required to achieve any significant degree of GAT1 C74 labeling, which made it difficult to carry out a thorough biophysical characterization of the labeled transporter.

With the long-term goal of utilizing endogenous, extracellularly oriented, solvent-exposed cysteine residues of GAT1 for specific and targeted transporter labeling in the *Xenopus laevis* oocyte plasma membrane, we set out to fully characterize the functional consequences of sulfhydryl modification of GAT1 with the membrane-impermeant sulfhydryl reagent [2-(trimethylammonium)ethyl]methanethiosulfonate (MTSET). Our data suggest that C74 is very likely the only functionally sensitive residue labeled by MTSET and that such labeling renders the transporter nonfunctional as well as electrically silent with respect to all known electrophysiological assays of transporter function (steady-state and presteady-state measurements). Labeling in the presence of valproate, an anion that specifically interacts with GAT1 and increases the rate of Na^+^/Cl^−^/GABA cotransport (Whitlow et al. 2003), significantly increases the accessibility of C74 to the extracellular fluid and, hence, allows for rapid and complete labeling of all transporter copies at the cell surface. Our findings have established the optimal conditions for labeling GAT1 in the plasma membrane and set the stage for future structure–function studies.

## Experimental Procedures

### Expression of Wild-type (WT) and Mutant GAT1 in *X. laevis* Oocytes

Stage V–VI *X. laevis* oocytes were injected with 50 ng of complementary RNA (cRNA) for human GAT1 (SLC6A1) (Nelson et al. 1990; Chen et al. 2004) or the mutant GAT1 C74A. The GAT1 C74A mutant was generated by polymerase chain reaction site-directed mutagenesis using the QuikChange Lightning Site-Directed Mutagenesis Kit (Agilent Technologies, La Jolla, CA). The sense (5′ GG TTC CCC TAT CTC GCC GGG AAA AAT GGT GGG 3′) and antisense (3′ CC AAG GGG ATA GAG CGG CCC TTT TTA CCA CCC 5′) mutagenic primers were synthesized by Retrogen (San Diego, CA); underlined bases denote the mutated codon. Following site-directed mutagenesis, the entire coding region of the plasmid containing GAT1 C74A was sequenced (Retrogen) to confirm the proper introduction of the mutation. cRNA for (WT) GAT1, or GAT1 C74A, was synthesized in vitro using T7 RNA polymerase (mMessage mMachine T7 Kit; Applied Biosystems/Ambion, Austin, TX). After cRNA injection, oocytes were maintained in Barth’s medium (in mM: 88 NaCl, 1 KCl, 0.33 Ca(NO_3_)_2_, 0.41 CaCl_2_, 0.82 MgSO_4_, 2.4 NaHCO_3_ and 10 HEPES [pH 7.4] as well as 50 μg/ml gentamicin, 100 μg/ml streptomycin and 100 U/ml penicillin) at 18 °C for up to 14 days until used in experiments. Unless otherwise indicated, all experiments were performed at 21 ± 2 °C.

### Experimental Solutions and Reagents

Unless otherwise indicated, experiments were performed in NaCl buffer containing (in mM) 100 NaCl, 2 KCl, 1 CaCl_2_, 1 MgCl_2_ and 10 HEPES (pH 7.4). In experiments which required Na^+^-free conditions, NaCl was isosmotically replaced, depending on the experimental protocol, with choline-Cl, LiCl, KCl, CsCl or tetraethylammonium-chloride. In experiments which required Cl^−^-free conditions, NaCl was isosmotically replaced with Na-[2-(*N*-morpholino) ethanesulfonic acid], Na-gluconate or Na-(2-propylpentanoic acid) (Na-valproate). In chloride-free buffers, KCl, CaCl_2_ and MgCl_2_ were replaced with their corresponding gluconate salts. The buffers used for solutions at pH 5.0 and 9.0 were MES and *N*-tris(hydroxymethyl)methyl-3-aminopropanesulfonic acid, respectively. GABA, 1-(4,4-diphenyl-3-butenyl)-3-piperidinecarboxylic acid (SKF-89976A), 1-[2-[[(diphenylmethylene)imino]oxy]ethyl]-1,2,5,6-tetrahydro-3-pyridinecarboxylic acid (NO-711), MTSET, 2-[(methylsulfonyl)thio]ethanesulfonic acid (MTSES, sodium salt), *N*-ethylmaleimide (NEM), tetramethylrhodamine-6-maleimide (TMR6M) and/or dithiothreitol (DTT) was added to the experimental solution as indicated. Stock solutions of sulfhydryl reagents (MTSET, MTSES, NEM and TMR6M) were stored in nanopure water (MTSET, MTSES), absolute ethanol (NEM) or dimethyl sulfoxide (TMR6M) at −20 °C and diluted in the experimental solution immediately before use. DTT was prepared from the solid material immediately before use. MTSET and MTSES were purchased from Toronto Research Chemicals (Toronto, ON, Canada) or Anatrace (Maumee, OH). TMR6M was purchased from Invitrogen (Carlsbad, CA). [^3^H]-GABA was obtained from GE Healthcare (Piscataway, NJ). All other reagents were purchased from Fisher Scientific (Pittsburgh, PA) or Sigma (St. Louis, MO).

### Electrophysiological Measurements and Data Analysis

The two-microelectrode voltage-clamp technique was used for the recording of whole-cell transporter-mediated currents. Oocytes were voltage-clamped at the indicated membrane potential (*V*_m_) using the Warner oocyte clamp (OC-725C; Warner Instrument; Hamden, CT). In the experimental recording chamber, oocytes were initially stabilized in NaCl buffer, and the composition of the bath was changed as indicated. In all experiments, reference electrodes were connected to the experimental oocyte chamber via agar bridges (3 % agar in 3 M KCl). For continuous holding current measurements, currents were low pass-filtered at 1 Hz (LPF 8, Warner Instrument) and sampled at 10 Hz (pCLAMP 8.1; Axon Instruments, Union City, CA). The GABA-evoked current was obtained as the difference in steady-state current in the absence and presence of GABA and/or inhibitor. As the GAT1-mediated, GABA-evoked current is Na^+^- and Cl^−^-coupled (Loo et al. 2000), it is referred to as $I_{\text{NaCl}}^{\text{GABA}}$ (Gonzales et al. 2007).

The effects of substrate concentration ([GABA]_o_, [Na^+^]_o_ and [Cl^−^]_o_) on the steady-state kinetics were determined by nonlinear curve fitting of the induced currents (*I*) with Eq. 1:

1 $$ I = \frac{{I_{\max }^{S} .\left[ S \right]^{n} }}{{\left( {K_{0.5}^{S} } \right)^{n} + \left[ S \right]^{n} }} $$

where *S* is the substrate (GABA, Na^+^ or Cl^−^), $I_{\max }^{S}$ is the maximal substrate-induced current, $K_{0.5}^{S}$ is the substrate concentration at half $I_{\max }^{S}$ (half-maximal concentration) and *n* is the Hill coefficient. For kinetic characterization of Cl^−^ activation of the inward currents, an additional linear term was added to Eq. 1 in order to account for the nonzero baseline at zero Cl^−^ concentration (see Fig. 6g–i).

To examine the carrier-mediated presteady-state current transients, the pulse protocol consisted of voltage jumps (400 ms) from the holding voltage (−50 mV) to test voltages ranging from +80 to −130 mV in 15-mV steps. Unless otherwise indicated, voltage pulses were separated by an interval of at least 3 s in order to allow for complete relaxation of the OFF transients (see Sacher et al. 2002; Karakossian et al. 2005). Currents were low pass-filtered at 1 kHz and sampled at 12.5 kHz without averaging. To obtain the transporter presteady-state currents, at each *V*_m_, the total current for the ON transients, *I*(*t*), was fitted with Eq. 2:

2 $$ I\left( t \right) = I_{1} e^{{ - t/\tau_{1} }} + I_{2} e^{{ - t/\tau_{2} }} + I_{\text{SS}} $$

where *t* is time, $I_{1} e^{{ - t/\tau_{1} }}$ is the oocyte capacitive transient current with initial value *I*_1_ and time constant τ_1_, $I_{2} e^{{ - t/\tau_{2} }}$ is the transporter transient current with initial value *I*_2_ and time constant τ_2_ and *I*_ss_ is the steady-state current (Loo et al. 1993; Hazama et al. 1997). At each *V*_m_, the total transporter-mediated charge (*Q*) was obtained by integration of the transporter transient currents. The charge–voltage (*Q–V*) relations obtained were then fitted with a single Boltzmann function (Eq. 3):

3 $$ \frac{{Q - Q_{\text{hyp}} }}{{Q_{\text{NaCl}} }} = \frac{1}{{1 + e^{{\left[ {\frac{{ - z\delta F\left( {V_{\text{m}} - V_{0.5} } \right)}}{RT}} \right]}} }} $$

where *Q*_NaCl_ = *Q*_dep_ − *Q*_hyp_ (*Q*_dep_ and *Q*_hyp_ are *Q* at depolarizing and hyperpolarizing limits, respectively) and represents the maximum charge moved (i.e., *Q*_max_), *z* is the apparent valence of movable charge, δ is the fraction of the membrane electric field traversed by the charge, *V*_0.5_ is the *V*_m_ for 50 % charge movement, *F* is Faraday’s constant, *R* is the gas constant and *T* is the absolute temperature.

The inhibition experiments involved GAT1-specific competitive inhibitors of GABA transport (SKF-89976A and NO-711). Data for the inhibition experiments were fitted with Eq. 4 (Krause and Schwarz 2005; Matthews et al. 2009):

4 $$ \frac{I}{{I_{ \max } }} = \frac{{[{\text{GABA}}]}}{{K_{0.5}^{\text{GABA}} \times \left( {1 + \frac{[B]}{{K_{i}^{B} }}} \right) + [{\text{GABA}}]}} $$

where *I*_max_ is the maximum current evoked by a saturating concentration of GABA in the absence of the blocker (*B*, here SKF-89976A or NO-711), *I* is the evoked current in the presence of the indicated concentrations of GABA and blocker (*B*), $K_{0.5}^{\text{GABA}}$ is the GABA concentration at which *I* is half of *I*_max_ (25 μM at −50 mV) (Gonzales et al. 2007) and $K_{i}^{B}$ is the blocker concentration at which *I* is 50 % of *I*_max_ (apparent half-inhibition constant).

**Fig. 1:**
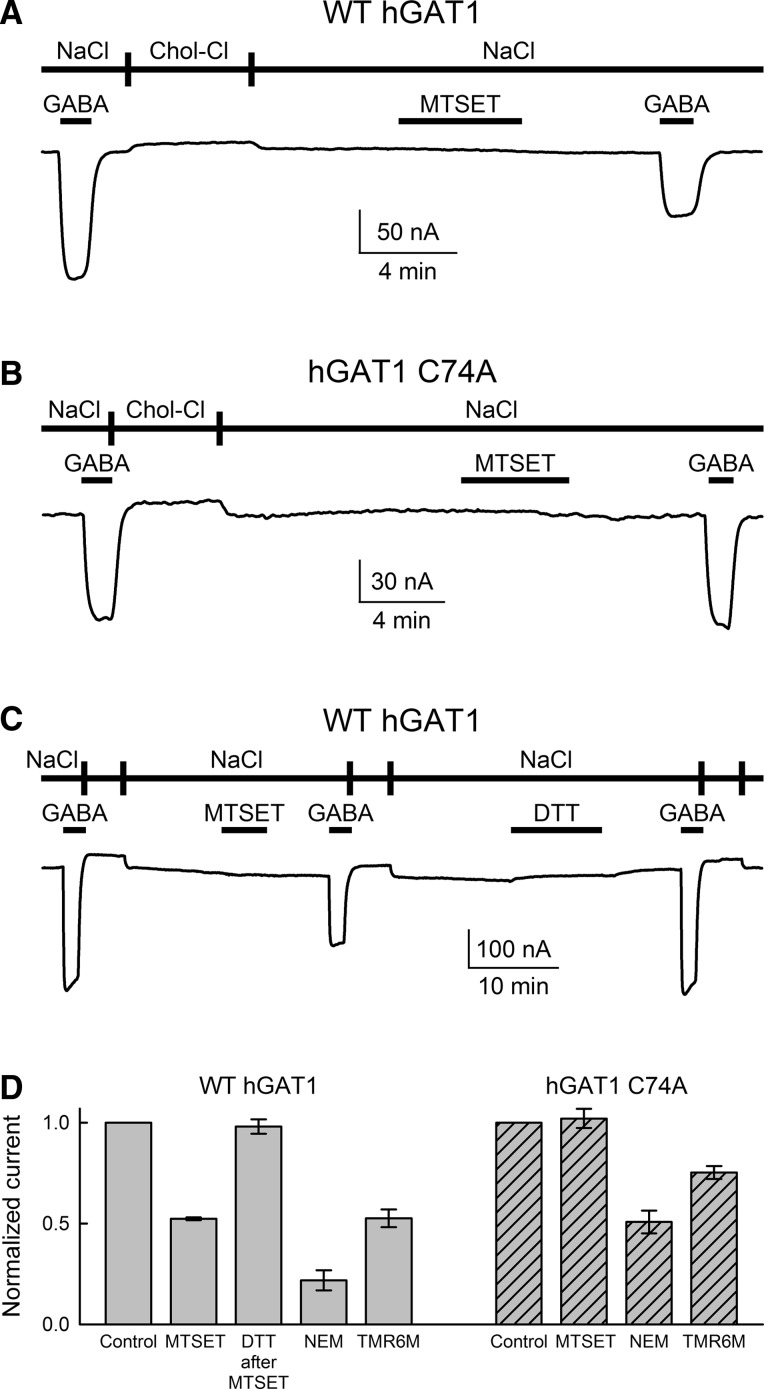
WT GAT1, but not GAT1 C74A, is sensitive to membrane-impermeant sulfhydryl reagents. **a** A representative GABA-evoked (500 μM) current trace is shown for WT GAT1 before and after labeling with the membrane-impermeant sulfhydryl reagent MTSET. Labeling of GAT1 with MTSET led to ~50 % reduction in the GABA-evoked current. The membrane potential (*V*_m_) was −50 mV throughout the experiment. Labeling was carried out at 1 mM MTSET for 5 min in NaCl buffer bathing the oocyte (at 21 ± 2 °C, pH 7.4). **b** Similar to WT GAT1, GAT1 C74A mediates Na^+^-dependent and Cl^−^-facilitated GABA transport (see Fig. 6). Exposure of GAT1 C74A to MTSET (1 mM for 5 min) had no effect on the magnitude of the GABA-evoked (500 μM) current. **c** Sulfhydryl modification of WT GAT1 with MTSET was completely reversed with DTT. Labeling with MTSET was carried out as in (**a**), and DTT was applied at 12 mM for 10 min in NaCl buffer. **d** Summary of data collected from four or more oocytes expressing WT GAT1 or GAT1 C74A. For each experimental condition, the GABA-evoked current obtained after labeling with MTSET, NEM or TMR6M (all at 1 mM for 5 min at −50 mV) was normalized to that prior to sulfhydryl modification in the same cell. Reported values represent the mean ± SE from four or more oocytes. Note that GAT1 C74A is insensitive to the membrane-impermeant MTSET but sensitive to the membrane-permeant NEM. GAT1 C74A was also sensitive to TMR6M

**Fig. 2:**
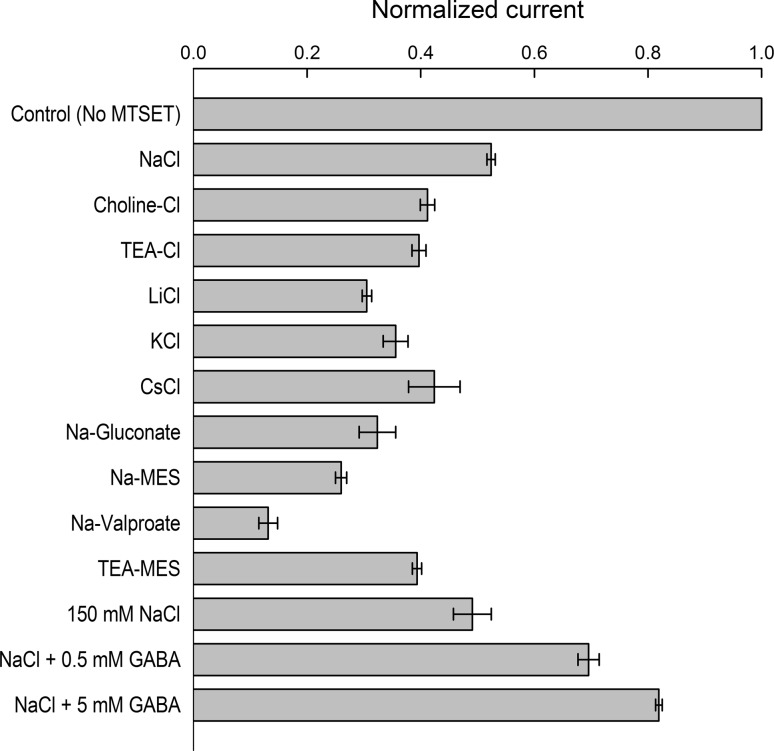
Effect of buffer composition on MTSET labeling of WT GAT1. WT GAT1 was labeled with MTSET in the indicated extracellular bathing solution, in which Na^+^ and/or Cl^−^ of the NaCl buffer had been isosmotically replaced by another cation or anion, respectively. The experimental protocol was similar to that shown in Fig. 1 (5-min exposure to 1 mM MTSET at −50 mV, 21 ± 2 °C, pH 7.4) with the exception that sulfhydryl modification was carried out in the indicated buffer. The GABA-evoked (500 μM in NaCl buffer) current after MTSET modification was normalized to that obtained in the same cell before exposure to MTSET. Reported values represent the mean ± SE from three or more oocytes

**Fig. 3:**
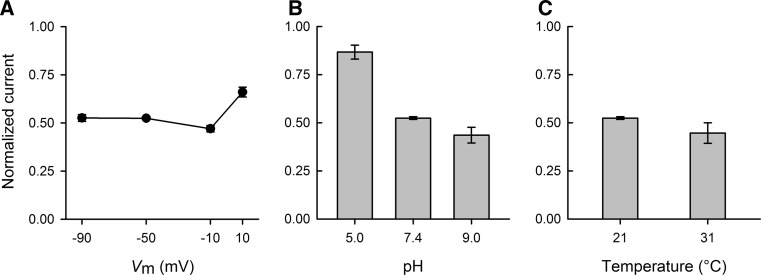
Effect of membrane potential, pH and temperature on MTSET labeling of WT GAT1. The GABA-evoked (500 μM at −50 mV) current mediated by WT GAT1 was measured before and after MTSET labeling (1 mM for 5 min) at different membrane potential values (**a**), extracellular pH values (**b**) and experimental temperatures (**c**). In all experiments, labeling was carried out in NaCl buffer. Labeling was performed at the indicated membrane potential values for the experiments of **a** and at −50 mV for the experiments of (**b**) and (**c**). Labeling was performed at pH 7.4 for the experiments of (**a**) and (**c**) and at the indicated values for the experiments of (**b**). Labeling was performed at 21 °C for the experiments of (**a**) and (**b**) and at the indicated values for the experiments of (**c**). The GABA-evoked current after MTSET modification was normalized to that obtained in the same cell before exposure to MTSET. Reported values represent the mean ± SE from three or more oocytes

**Fig. 4:**
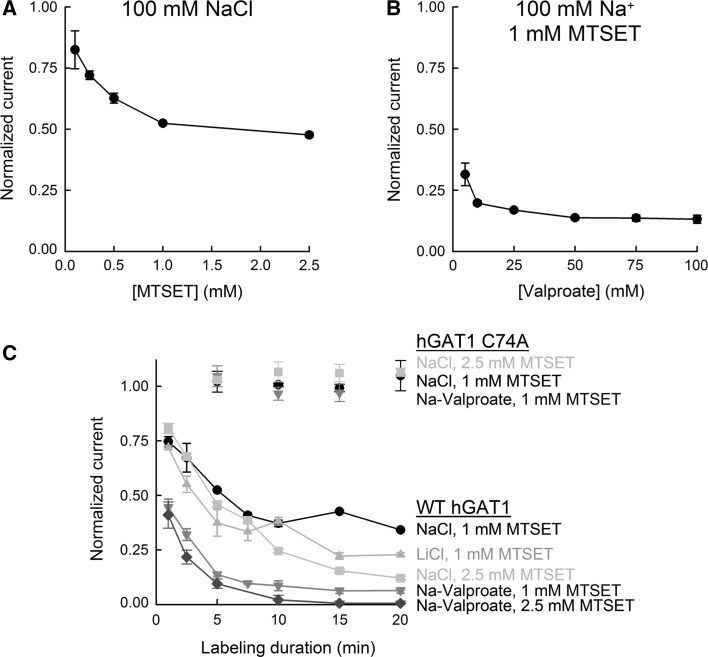
Effect of MTSET concentration and labeling duration on sulfhydryl modification of WT GAT1. **a** Sulfhydryl modification of WT GAT1 was carried out for 5 min in NaCl buffer in the presence of the indicated concentration of MTSET. **b** Sulfhydryl modification of WT GAT1 was carried out at 1 mM MTSET for 5 min in the presence of the indicated concentration of valproate. [Na^+^] = 100 mM. **c** Duration of labeling with 1 or 2.5 mM MTSET was varied (up to 20 min) in NaCl, LiCl or Na-valproate buffer for WT GAT1 or GAT1 C74A. Note that under all conditions GAT1 C74A was functionally insensitive to MTSET exposure. See text for the second-order rate constants for MTSET labeling of WT GAT1 under different conditions. In all experiments, labeling was carried out at −50 mV. In all experiments, the GABA-evoked (500 μM in NaCl buffer at −50 mV) current after MTSET modification was normalized to that obtained in the same cell before exposure to MTSET. Reported values represent the mean ± SE from three or more oocytes

To determine the effect of sulfhydryl modification on the ratio of GAT1-mediated charge flux to GABA flux across the plasma membrane, uptake experiments were performed under voltage clamp in individual control oocytes and oocytes expressing WT GAT1 (Eskandari et al. 1997; Forster et al. 1999; Loo et al. 2000; Sacher et al. 2002; Whitlow et al. 2003; Karakossian et al. 2005; Gonzales et al. 2007; Matthews et al. 2009). The membrane potential was held at the indicated value (−50 mV), and the holding current was continuously monitored. Oocytes were initially incubated in NaCl buffer until a baseline was established. GABA (500 μM) and [^3^H]-GABA (20 nM) were added to the perfusion solution for 5–10 min. At the end of the incubation period, GABA and the isotope were removed from the perfusion solution until the holding current returned to baseline. Oocytes were removed from the experimental chamber, washed in ice-cold choline-Cl buffer and solubilized in 10 % sodium dodecyl sulfate (SDS). Oocyte [^3^H]-GABA content was determined in a liquid scintillation counter (LS 6500; Beckman, Fullerton, CA). Net inward charge was obtained from the time integral of the GABA-evoked inward current and correlated with GABA influx in the same cell. GABA uptake under voltage clamp was also performed in oocytes expressing WT GAT1 after a 5-min exposure to 1 mM MTSET in NaCl buffer. This treatment reduces the GAT1-mediated, GABA-evoked current by approximately 50 % (see Figs. 1, 2, 3, 4, 7). In all experiments, the GAT1-mediated GABA uptake was obtained by subtracting endogenous GABA uptake in control cells from the same batch that were subjected to the same experimental condition as GAT1-expressing cells. Depending on the level of GAT1 expression in the plasma membrane as well as the duration of GABA exposure (5–10 min), the magnitude of endogenous GABA uptake was 0.5–5 % of the total GABA uptake (endogenous plus GAT1-mediated uptake).

Where sample sizes are indicated (*n*), they refer to the number of oocytes in which the experiments were repeated. Reported errors represent the standard error of the mean obtained from data from several oocytes.

## Results

### Labeling with the Membrane-Impermeant Sulfhydryl Reagent MTSET Leads to Functional Modification of WT GAT1 but not the Mutant GAT1 C74A

Figure 1 shows the basic experimental approach used to assess the effect of sulfhydryl modification on the GABA-evoked current mediated by GAT1 ($I_{\text{NaCl}}^{\text{GABA}}$). $I_{\text{NaCl}}^{\text{GABA}}$ is proportional to the number of functional transporters in the plasma membrane and is directly related to Na^+^/Cl^−^/GABA cotransport across the plasma membrane (Loo et al. 2000; Gonzales et al. 2007; Matthews et al. 2009). It is, therefore, a good assay of GAT function. In a typical experiment, $I_{\text{NaCl}}^{\text{GABA}}$ was measured in NaCl buffer (plus 500 μM GABA) before and after exposure to MTSET. In the experiments of Fig. 1, MTSET labeling was carried out in NaCl buffer at 1 mM for 5 min (at 21 ± 2 °C, *V*_m_ = −50 mV, pH 7.4). The evoked current obtained after MTSET exposure was normalized to that before MTSET and was 52 ± 1 % of the original value (*n* = 4) (Fig. 1a, d). Treatment with the membrane-impermeant MTSES (at 1 mM for 5 min at 21 ± 2 °C, *V*_m_ = −50 mV, pH 7.4) reduced $I_{\text{NaCl}}^{\text{GABA}}$ by only 22 ± 2 % (*n* = 3, data not shown). Therefore, we focused on the effect of MTSET for the remaining experiments of this study. In all subsequent experiments, where normalized current values are reported, they always refer to measurements obtained in NaCl buffer (500 μM GABA at 21 ± 2 °C, *V*_m_ = −50 mV, pH 7.4). However, the labeling conditions were altered depending on the experimental objective. Unless otherwise stated, the labeling conditions consisted of 1 mM MTSET for 5 min in NaCl buffer at 21 ± 2 °C, *V*_m_ = −50 mV, pH 7.4.

Similar to WT GAT1, GAT1 C74A mediates Na^+^-dependent and Cl^−^-facilitated GABA transport (see Fig. 6). Exposure of GAT1 C74A to MTSET (1 mM for 5 min) had no effect on the magnitude of the GABA-evoked current (Fig. 1b, d). This result suggests that C74 is very likely the only functionally significant cysteine residue that is exposed to the extracellular fluid (and hence accessible to the membrane-impermeant MTSET).

The reduction in WT GAT1 $I_{\text{NaCl}}^{\text{GABA}}$ caused by MTSET sulfhydryl modification was completely reversible with DTT. Following DTT treatment (12 mM for 10 min), $I_{\text{NaCl}}^{\text{GABA}}$ returned to 98 ± 3 % of its original level before MTSET labeling (*n* = 5) (Fig. 1c, d). Treatment of WT GAT1 with DTT, either before or during exposure to GABA, was without any effect on the GABA-evoked current. In the copresence of 500 μM GABA and 10 mM DTT, $I_{\text{NaCl}}^{\text{GABA}}$ was 98 ± 2 % of that measured in the same cell in the absence of DTT (*n* = 3) (not shown).

The above results suggest that any functional consequence of sulfhydryl modification of WT GAT1 by MTSET very likely occurred at position C74. NEM, a membrane-permeant thiol modification reagent, led to a significant reduction in $I_{\text{NaCl}}^{\text{GABA}}$ mediated by both WT GAT1 (21 ± 5 %, *n* = 4) and GAT1 C74A (51 ± 6 %, *n* = 5) (Fig. 1d). These results further suggest that C74 is very likely the only functionally sensitive cysteine residue exposed to the extracellular fluid. TMR6M, a fluorescent probe commonly used for labeling membrane proteins at solvent-exposed cysteine residues, reduced $I_{\text{NaCl}}^{\text{GABA}}$ mediated by WT GAT1 to 53 ± 4 % (*n* = 3) and that mediated by GAT1 C74A to 75 ± 3 % (*n* = 6) of control levels (Fig. 1d).

As the long-term objective of this project is to utilize extracellularly exposed cysteine residues to label and quantify the number of transporter copies in the plasma membrane, we carried out a complete characterization of labeling WT GAT1 with MTSET with the goal of identifying experimental conditions that drive the labeling reaction to completion (i.e., 100 % labeling of transporter copies in the plasma membrane). Understanding these conditions would then allow us to pursue future labeling and quantification studies.

### The Inhibitory Effect of MTSET is Dependent on the Composition of the Labeling Buffer

GABA transport by the GATs is Na^+^- and Cl^−^-coupled, and indeed, Na^+^ and Cl^−^ interaction with the transporter leads to conformational changes (Golovanevsky and Kanner 1999; Lu and Hilgemann 1999b; Li et al. 2000; Zomot and Kanner 2003; Meinild et al. 2009). Therefore, we explored the possibility that the ionic composition of the buffer in which the MTSET exposure takes place can potentially enhance the solvent accessibility of C74, thus increasing the degree of labeling. To accomplish this, we investigated the degree of MTSET labeling when Na^+^ and/or Cl^−^ was isosmotically replaced with various cations/anions in the labeling buffer, keeping all other labeling conditions constant (1 mM MTSET for 5 min at 21 ± 2 °C, *V*_m_ = –50 mV, pH 7.4).

Labeling with MTSET in the standard NaCl conditions reduced $I_{\text{NaCl}}^{\text{GABA}}$ to 52 ± 1 % of control levels (Figs. 1, 2). When Na^+^ in the labeling buffer was replaced with other cations such as choline, TEA, Cs^+^, K^+^ or Li^+^, there was only a marginal enhancement of the inhibitory effect of MTSET (Fig. 2). The largest effect of Na^+^ replacement was seen with Li^+^ (Fig. 2). Similarly, replacing Cl^−^ in the labeling buffer with anions such as MES or gluconate only modestly enhanced the inhibitory effect of MTSET. Neither replacement of both Na^+^ and Cl^−^ in the labeling buffer (with TEA and MES, respectively) nor increasing the NaCl concentration to 150 mM led to any significant increase in the degree of MTSET labeling (Fig. 2). Notably, labeling with MTSET when Cl^−^ was replaced with valproate resulted in significantly enhanced efficacy of the MTSET-dependent reduction in $I_{\text{NaCl}}^{\text{GABA}}$ (13 ± 2 % of control, *n* = 4). On the other hand, the presence of GABA during MTSET treatment protected against transporter labeling (Fig. 2). Addition of 0.5 and 5 mM GABA to the labeling buffer reduced $I_{\text{NaCl}}^{\text{GABA}}$ to only 70 ± 3 % (*n* = 3) and 82 ± 2 % (*n* = 3) of control levels prior to MTSET labeling (Fig. 2).

### Changes in Membrane Potential, pH and Temperature Do Not Alter the Effect of MTSET

GAT activity is also known to be altered by changes in membrane potential (Mager et al. 1993, 1996; Lu and Hilgemann 1999a, b; Gonzales et al. 2007), pH (Grossman and Nelson 2002, 2003) and temperature (Lu and Hilgemann 1999a; Binda et al. 2002; Gonzales et al. 2007; Matthews et al. 2009). Therefore, we examined the influence of these factors on the degree of MTSET labeling of GAT1. In the experiments of Fig. 3, membrane potential (*V*_m_), pH and temperature were independently perturbed during the MTSET labeling period, while all other parameters were held constant according to our standard labeling conditions: 1 mM MTSET for 5 min at 21 ± 2 °C, *V*_m_ = −50 mV and pH 7.4. Interestingly, changes in the membrane potential or pH of the labeling buffer or in temperature had little to no effect on the efficacy of MTSET labeling (Fig. 3). The apparent reduction in MTSET labeling at lower pH values (Fig. 3b) is unlikely to be a direct conformational effect on the transporter (see “Discussion” Section).

### MTSET Labeling Conditions for Complete Elimination of GAT1-Mediated GABA Transport

In further pursuit of the optimal experimental conditions under which MTSET labeling of WT GAT1 would approach completion, we examined the effect of MTSET concentration (Fig. 4a) and labeling duration (Fig. 4c) on GAT1-mediated, GABA-evoked current. The concentration dependence of MTSET labeling in NaCl buffer is shown in Fig. 4a. Keeping all other labeling conditions constant, increasing the concentration of MTSET from 1 to 2.5 mM slightly decreased the normalized $I_{\text{NaCl}}^{\text{GABA}}$ current from 52 ± 1 % (*n* = 4) to 48 ± 1 % (*n* = 4) of values prior to MTSET labeling. The estimated *K*_i_ for MTSET inhibition of $I_{\text{NaCl}}^{\text{GABA}}$ was 1.6 ± 0.2 mM. Inclusion of valproate in the labeling buffer significantly enhanced the labeling efficacy of MTSET (see Fig. 2). The half-maximal concentration for the valproate-induced enhancement of MTSET labeling was estimated to be 16 ± 3 mM (*n* = 3) (Fig. 4b).

Increasing the MTSET incubation time in NaCl buffer to 20 min decreased the GAT1-mediated, GABA-evoked current to 34 ± 1 % at 1 mM MTSET (*n* *=* 3) and 12 ± 1 % at 2.5 mM MTSET (*n* *=* 3) (Fig. 4c). Similarly, MTSET incubation in LiCl buffer for up to 20 min failed to lead to 100 % elimination of $I_{\text{NaCl}}^{\text{GABA}}$ (Fig. 4c). However, labeling with 2.5 mM MTSET in the presence of Na-valproate for ≥10 min brought about complete elimination of GAT1-mediated GABA transport (Fig. 4c). The second-order rate constants (Zhang and Karlin 1997) for MTSET labeling of WT GAT1 were determined to be 0.79 ± 0.21 M^−1^ s^−1^ (1 mM MTSET in NaCl), 2.0 ± 0.2 M^−1^ s^−1^ (2.5 mM MTSET in NaCl), 1.2 ± 0.3 M^−1^ s^−1^ (1 mM MTSET in LiCl), 3.7 ± 0.4 M^−1^ s^−1^ (1 mM MTSET in Na-valproate) and 6.4 ± 0.5 M^−1^ s^−1^ (2.5 mM MTSET in Na-valproate).

Importantly, $I_{\text{NaCl}}^{\text{GABA}}$ mediated by GAT1 C74A was not inhibited by MTSET under any labeling condition (Fig. 4c). Taken together, these results further support the notion that C74 is only partially exposed to the extracellular fluid and that ionic interactions with the transporter can enhance the solvent accessibility of this residue (Yu et al. 1998; Meinild et al. 2009).

**Fig. 5:**
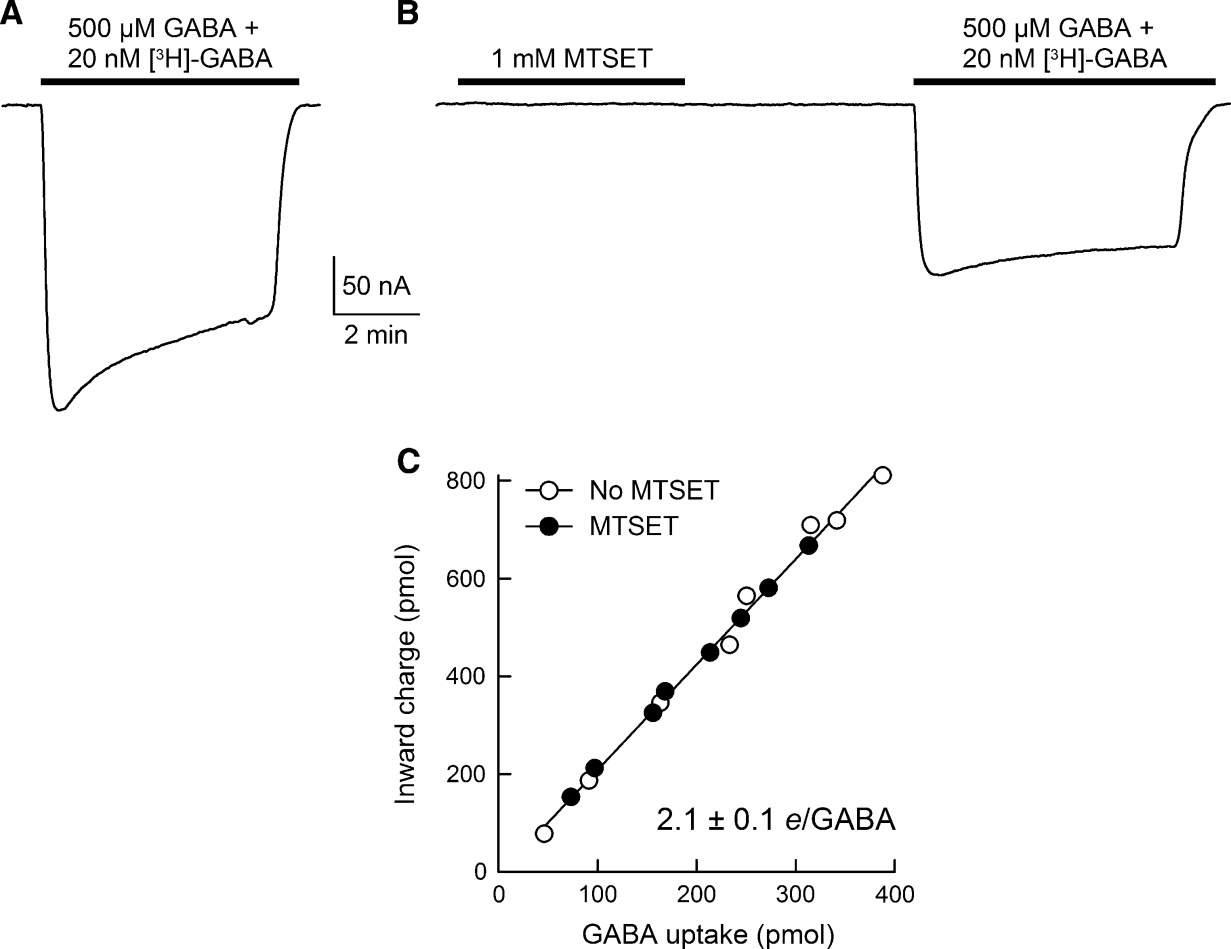
Sulfhydryl modification of WT GAT1 with MTSET does not alter the ion/GABA transport coupling ratio. GABA-uptake experiments were performed under voltage clamp without MTSET treatment (**a**) or after labeling with 1 mM MTSET for 5 min (**b**). *V*_m_ = −50 mV. **c** Cells expressing GAT1 were exposed to 500 μM GABA and 20 nM [^3^H]-GABA for 5–10 min. After washout of GABA and isotope, cells were solubilized in 10 % SDS and intracellular GABA content was determined using a liquid scintillation counter. In the same cell, the net inward charge flux was obtained from the time integral of the GABA-evoked current trace. The ratio of charge flux to GABA flux (i.e., net positive charges per GABA, *e*/GABA) was 2.1 ± 0.1 whether or not the cell was exposed to MTSET (*n* = 8 and 8). The *smooth line* in (**c**) is a linear regression through all data points

**Fig. 6:**
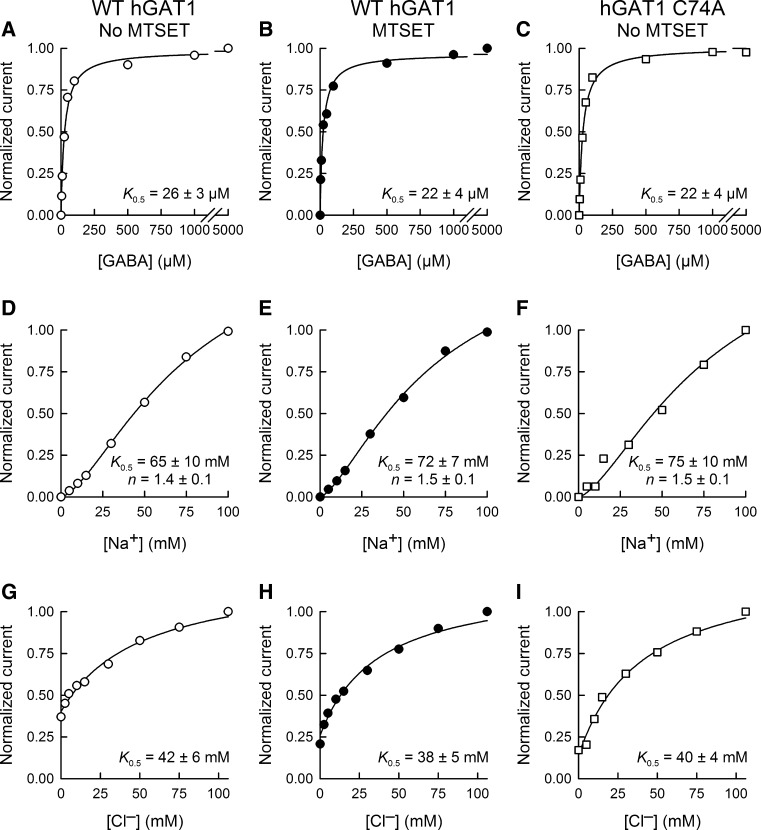
Steady-state kinetic parameters of WT GAT1 before and after sulfhydryl modification with MTSET. Representative GABA (**a**–**c**), Na^+^ (**d**–**f**) and Cl^−^ (**g**–**i**) steady-state kinetic curves are shown for WT GAT1 before (*left panels*) and after (*middle panels*) treatment with MTSET (1 mM for 5 min at −50 mV) as well as for GAT1 C74A without MTSET treatment (*right panels*). The half-maximal concentration values (*K*_0.5_) for GABA, Na^+^ and Cl^−^ were similar for WT GAT1 and GAT1 C74A. The Hill coefficient values for Na^+^ and Cl^−^ activation of the inward currents were also similar for WT GAT1 and GAT1 C74A. Moreover, MTSET exposure had no effect on WT GAT1 steady-state kinetic parameters. *V*_m_ = −50 mV. For GABA kinetic experiments (**a–c**), [Na^+^]_0_ was 100 mM and [Cl^−^] was 106 mM. For sodium kinetic experiments (**d**–**f**), [Cl^−^] was 106 mM and [GABA]_0_ was 5 mM. For chloride kinetic experiments (**g**–**i**), [Na^+^]_0_ was 100 mM and [GABA]_0_ was 5 mM. The *smooth lines* represent fits of the experimental data with Eq. 1 (see “Experimental Procedures” Section). Reported values represent the mean ± SE from three or more oocytes

**Fig. 7:**
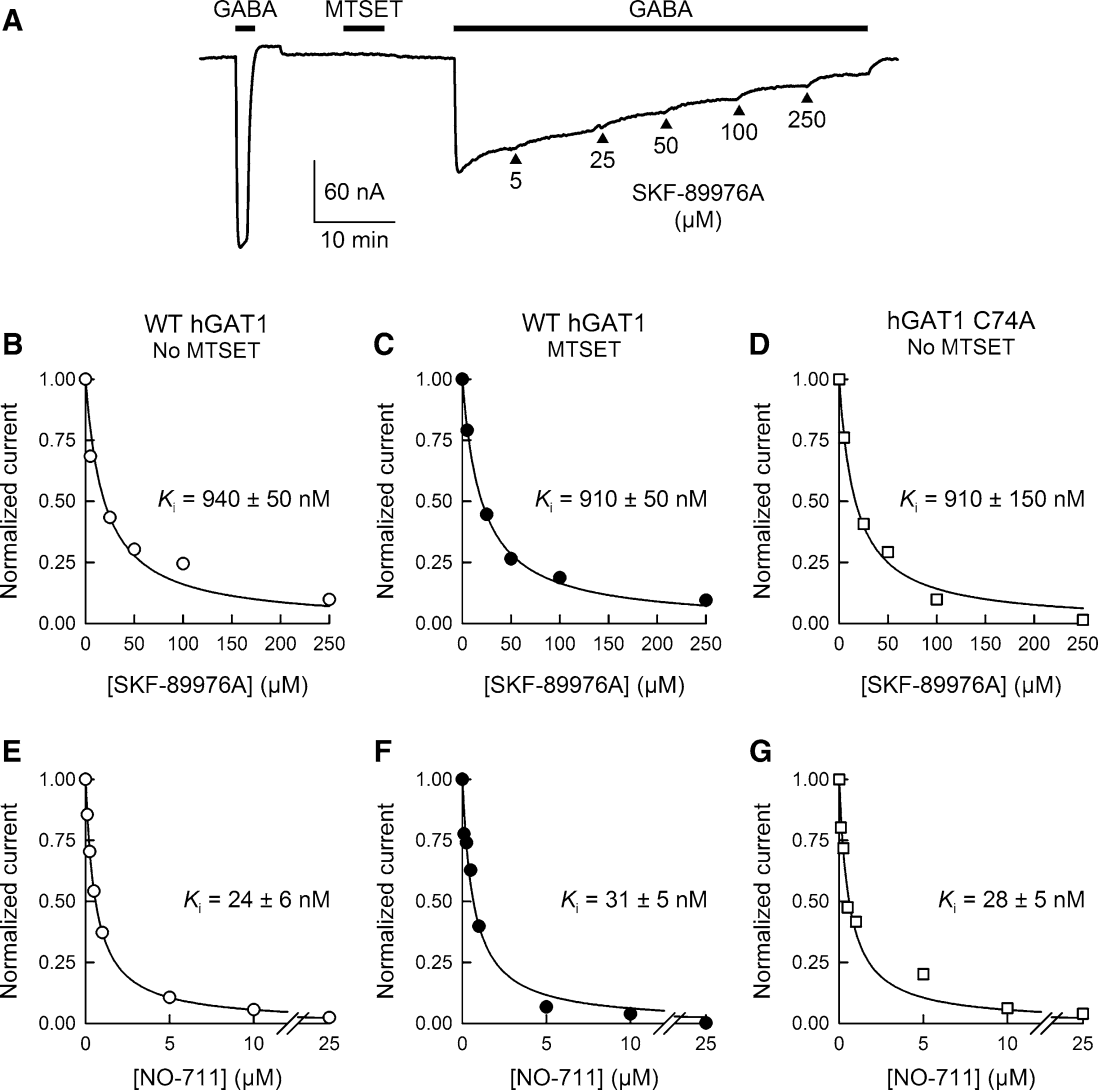
Sulfhydryl modification with MTSET has no effect on WT GAT1 sensitivity to transport inhibitors. **a** A representative WT GAT1 current trace is shown for inhibition of the GABA-evoked (500 μM at −50 mV) current with SKF-89976A (a specific inhibitor of GAT1) following sulfhydryl modification with MTSET (1 mM for 5 min at −50 mV). Similar to that shown in this panel, inhibition kinetics experiments were performed with SKF-89976A or NO-711 for WT GAT1 without (**b**, **e**) or with (**c**, **f**) prior exposure to MTSET (1 mM for 5 min at −50 mV) as well as for GAT1 C74A without MTSET treatment (**d**, **g**). Sulfhydryl modification of WT GAT1 had no effect on the *K*_i_ values for SKF-89976A or NO-711. Moreover, the *K*_i_ values for SKF-89976A (**d**) and NO-711 (**g**) inhibition of GAT1 C74A GABA-evoked currents were not different from those of WT GAT1. The *smooth lines* in (**b**–**g**) represent fits of the experimental data with an equation for competitive inhibition at a single binding site (Eq. 4, see “Experimental Procedures” section). Reported *K*_i_ values represent the mean ± SE from three or more oocytes

**Fig. 8:**
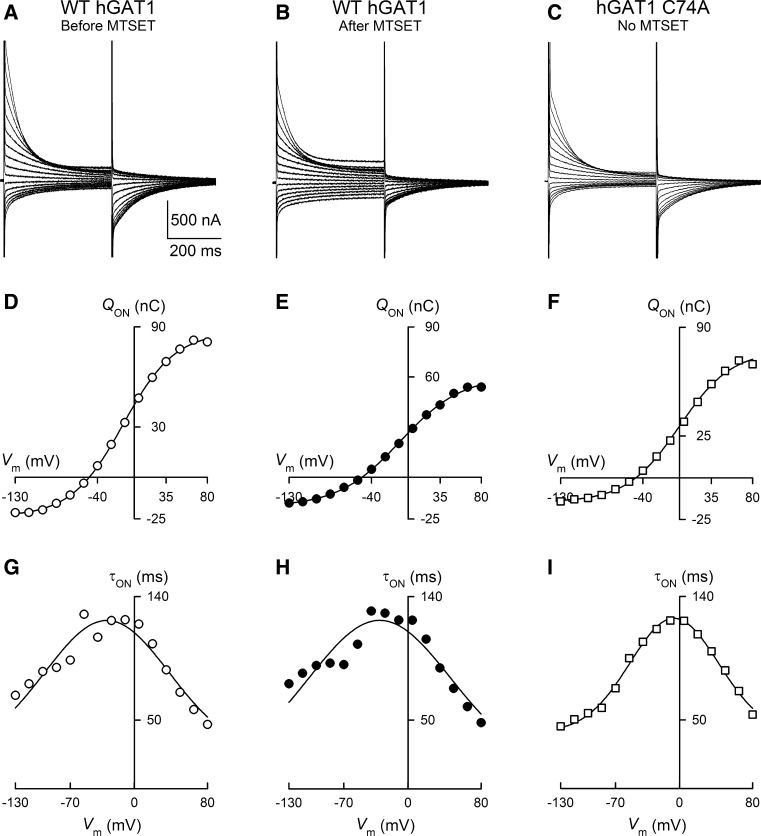
Presteady-state charge movements of WT GAT1 before and after sulfhydryl modification with MTSET. **a**–**c** Representative current relaxations are shown in response to 400-ms voltage pulses from +80 to −130 mV for WT GAT1 before (**a**) and after (**b**) treatment with MTSET (1 mM for 5 min) as well as for GAT1 C74A without MTSET treatment (**c**). Holding potential was −50 mV. **d**–**f** For each evoked current trace, time integration of the ON presteady-state currents yielded the total charge moved (*Q*_ON_) and, when plotted as a function of the test voltage, the *Q*–*V* relationship for WT GAT1 before (**d**) and after (**e**) labeling with MTSET as well as for GAT1 C74A (**f**). The *smooth lines* in (**d**–**f**) represent the fit of the data with a Boltzmann function (Eq. 3, see “Experimental Procedures” section). **g**–**i** Presteady-state currents monoexponentially decay to the zero level. The time constant plotted as a function of the test voltage yielded the τ–*V* relationship. The *smooth lines* in (**g**–**i**) represent the fit of the data with a gaussian function (Sacher et al. 2002). While MTSET treatment led to a reduction in the total charge moved (compare **d** and **e**), it had no effect on the midpoint of the *Q*–*V* relationship (*V*_0.5_) and no effect on the relaxation time constants (compare **g** and **h**). The presteady-state parameters of GAT1 C74A were not different from those of WT GAT1. See text for additional details

**Fig. 9:**
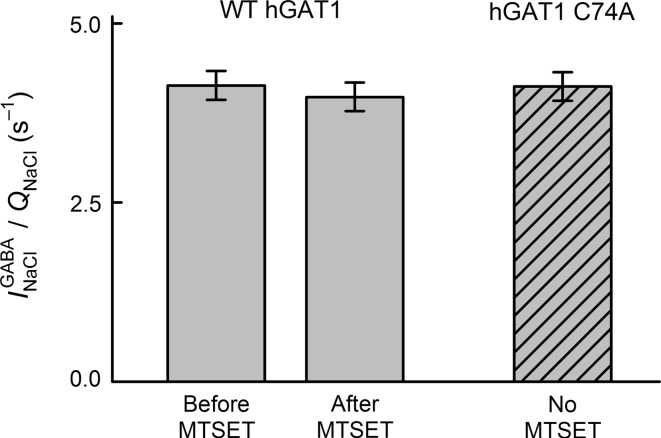
Sulfhydryl modification with MTSET has no effect on WT GAT1 turnover rate. The ratio of $I_{\text{NaCl}}^{\text{GABA}}$ to *Q*_NaCl_ is used as an index of the transporter turnover rate (Gonzales et al. 2007). $I_{\text{NaCl}}^{\text{GABA}}$ represents the maximum GABA-evoked current and was measured at 5 mM GABA and −50 mV (in NaCl buffer). *Q*_NaCl_ represents the total charge moved in response to ON voltage pulses and was measured in NaCl buffer according to the procedures described for the experiments of Fig. 8. Sulfhydryl modification with MTSET had no effect on the ratio of $I_{\text{NaCl}}^{\text{GABA}}$ to *Q*_NaCl_ (4.1 ± 0.2 vs. 4.0 ± 0.2 s^−1^, *n* *=* 4 and 4). The ratio was also similar in GAT1 C74A (4.1 ± 0.2 s^−1^, *n* *=* 7)

Thus far, the results demonstrate that sulfhydryl modification of GAT1 at C74 leads to a reduction in the GABA-evoked current ($I_{\text{NaCl}}^{\text{GABA}}$). In order to better understand the mechanism by which MTSET labeling of WT GAT1 leads to a reduction in $I_{\text{NaCl}}^{\text{GABA}}$, we carried out a comprehensive characterization of the current that remains after partial labeling with 1 mM MTSET for 5 min (see Fig. 1). In principle, the reduction in $I_{\text{NaCl}}^{\text{GABA}}$ may be due to (1) a change in the transporter ion/GABA flux coupling ratio; (2) a significant increase in the apparent affinity constant (*K*_0.5_) values (i.e., reduction in the apparent affinity) for the transporter cosubstrates Na^+^, Cl^−^ and GABA; (3) a reduction in transporter turnover rate (i.e., the rate at which ion/GABA molecules are cotranslocated across the plasma membrane per unit time); or (4) a complete loss of GAT1 GABA transport activity when C74 is chemically modified by MTSET. The experiments of Figs. 5, 6, 7, 8 and 9 examine these four possibilities. In all of the following experiments, WT GAT1 was labeled with 1 mM MTSET for 5 min (at 21 ± 2 °C, *V*_m_ = −50 mV and pH 7.4) in order to achieve ~50 % reduction in $I_{\text{NaCl}}^{\text{GABA}}$ (see Figs. 1, 2, 3, 4). This “partial labeling” protocol allowed us to carry out a complete functional characterization of $I_{\text{NaCl}}^{\text{GABA}}$ after MTSET labeling.

### Sulfhydryl Modification of GAT1 C74 Does Not Alter GAT1-Mediated Charge/GABA Flux Ratio

Translocation of GABA by GAT1 across the plasma membrane is coupled to cotranslocation of Na^+^ and Cl^−^ (Radian and Kanner 1983; Keynan and Kanner 1988; Loo et al. 2000), and recent studies suggest that Na^+^/Cl^−^/GABA cotransport is a tightly coupled process (Gonzales et al. 2007; Matthews et al. 2009). To examine the possibility that MTSET modification of C74 leads to a change in the ion/GABA coupling ratio of GAT1, we performed GABA uptake under voltage-clamp experiments (Fig. 5). In these experiments, [^3^H]-GABA uptake was measured under voltage clamp in individual GAT1-expressing cells in the absence of MTSET labeling (Fig. 5a, c) or after labeling with 1 mM MTSET for 5 min (at 21 ± 2 °C, *V*_m_ = –50 mV and pH 7.4) (Fig. 5b, c). This experimental protocol yielded two measured parameters for each cell: (1) net GABA-evoked inward charge translocation during the recording period (charge flux) and (2) GABA uptake in the same cell (GABA flux) (Fig. 5c). The ratio of charge flux to GABA flux was 2.1 ± 0.1 whether or not the cell was exposed to MTSET (Fig. 5c).

### Sulfhydryl Modification of GAT1 C74 Does Not Alter Transporter Steady-State or Presteady-State Kinetic Parameters

To determine if MTSET modification of GAT1 C74 alters the transporter steady-state kinetic properties, we performed steady-state kinetic experiments for GABA, Na^+^ and Cl^−^ in WT GAT1 before and after labeling with MTSET (1 mM for 5 min in NaCl buffer) (Fig. 6). The results of these experiments show that the apparent affinity constants for GABA (26 ± 3 vs. 22 ± 4 μM, Fig. 6a, b), Na^+^ (66 ± 10 vs. 72 ± 7 mM, Fig. 6d, e) and Cl^−^ (42 ± 6 vs. 38 ± 5 mM, Fig. 6g, h) were not significantly different before and after partial labeling with 1 mM MTSET for 5 min. Moreover, the Hill coefficient for Na^+^ activation of the currents was the same before and after sulfhydryl modification of C74 (Fig. 6d, e). The apparent affinity constants of the mutant GAT1 C74A for GABA (22 ± 4 μM, Fig. 6c), Na^+^ (75 ± 10 mM, Fig. 6f) and Cl^−^ (40 ± 4 mM, Fig. 6i) were not significantly different from those of WT GAT1.

To further characterize the behavior of WT GAT1 after partial labeling with 1 mM MTSET for 5 min, we investigated the sensitivity of WT GAT1 to known transporter inhibitors before and after sulfhydryl modification (Fig. 7). The apparent half-inhibition constant values for SKF-89976A (940 ± 50 vs. 910 ± 50 nM, Fig. 7b, c) and NO-711 (24 ± 6 vs. 31 ± 5 nM, Fig. 7e, f) were not significantly different before and after sulfhydryl labeling with MTSET. Moreover, the apparent half-inhibition constant values of GAT1 C74A for SKF-89976A (910 ± 150 nM, Fig. 7d) and NO-711 (28 ± 5 nM, Fig. 7g) were similar to those of WT GAT1.

To examine the effect of MTSET labeling on WT GAT1 voltage-evoked presteady-state currents, 400-ms voltage pulses were applied, from a holding voltage of −50 mV to test voltages ranging from +80 to −130 mV in 15-mV steps, before (Fig. 8a) and after (Fig. 8b) labeling with 1 mM MTSET for 5 min. At each voltage, the evoked current transient was analyzed to obtain the GAT1-mediated charge moved (see “Experimental Procedures” Section), and the resulting presteady-state charge was plotted as a function of the test voltage to obtain the *Q*–*V* relationships (Fig. 8d, e). After labeling WT GAT1 with 1 mM MTSET for 5 min, *Q*_NaCl_ was reduced to 60 ± 8 % (*n* *=* 4) of that prior to MTSET treatment (compare Fig. 8d, e), suggesting a similar degree of loss of function as that seen with steady-state GABA-evoked current ($I_{\text{NaCl}}^{\text{GABA}}$) (see Figs. 1, 2, 3). Neither the midpoint of the *Q*–*V* relationship (*V*_0.5_, 25 ± 5 vs. 22 ± 6 mV) nor the apparent valence of the moveable charge (*z*δ, 0.9 ± 0.1 vs. 0.9 ± 0.1) was different after MTSET treatment (Fig. 8d, e). Furthermore, the ON and OFF relaxation time constants were not altered after MTSET treatment (Fig. 8g, h). Finally, the presteady-state kinetic properties of GAT1 C74A were similar to those of WT GAT1 (Fig. 8c, f, i).

### Sulfhydryl Modification of GAT1 C74 Does Not Alter Transporter Turnover Rate

Turnover rate is the rate at which ion/GABA molecules are cotranslocated across the plasma membrane per unit time. In a comprehensive characterization, the GAT1 turnover rate was determined to be 15 GABA molecules per second at 21 °C, −50 mV and physiological ion concentrations (Gonzales et al. 2007). When extrapolated to the physiological temperature of 37 °C, the turnover rate was estimated to be 79 s^−1^ (Gonzales et al. 2007). An important prerequisite to characterizing the turnover rate is the ratio of the maximum GABA-evoked current ($I_{\text{NaCl}}^{\text{GABA}}$) to the maximum presteady-state charge movements (*Q*_NaCl_). Both $I_{\text{NaCl}}^{\text{GABA}}$ and *Q*_NaCl_ are directly proportional to the total number of functional transporters in the cell plasma membrane (Gonzales et al. 2007). The ratio of $I_{\text{NaCl}}^{\text{GABA}}$ to *Q*_NaCl_ is directly proportional to the turnover rate and, therefore, can be used as an index of the transporter turnover rate (for details, see Gonzales et al. 2007). At any given membrane potential, the maximum GABA-evoked current ($I_{\text{NaCl}}^{\text{GABA}}$) is measured at steady state after application of GABA at a saturating concentration (≥5 mM). The maximum presteady-state charge movement (*Q*_NaCl_) is determined in the absence of GABA from analyzing presteady-state current transients evoked in response to step changes in the membrane potential (Sacher et al. 2002; Whitlow et al. 2003; Karakossian et al. 2005; Gonzales et al. 2007). The results of typical presteady-state charge movement experiments are shown in Fig. 8, and the ratio of $I_{\text{NaCl}}^{\text{GABA}}$ to *Q*_NaCl_ is shown in Fig. 9.

The ratio of $I_{\text{NaCl}}^{\text{GABA}}$ to *Q*_NaCl_ (i.e., index of turnover rate) for WT GAT1 was the same before and after sulfhydryl modification with 1 mM MTSET for 5 min (4.1 ± 0.2 vs. 4.0 ± 0.2 s^−1^, *n* *=* 4 and 4). Moreover, this ratio was similar for GAT1 C74A (4.1 ± 0.2 s^−1^, *n* *=* 7).

## Discussion

In anticipation of utilizing extracellularly oriented, solvent-exposed cysteine residues of GAT1 for labeling and quantifying the transporter in the plasma membrane, the primary objective of this study was to characterize the functional consequences of sulfhydryl modification of GAT1 with the membrane-impermeant sulfhydryl reagent MTSET. Our results show that exposure of the extracellular surface of WT GAT1 to MTSET resulted in a significant and proportional, yet reversible, decrease in both the steady-state GABA-evoked current as well as the presteady-state charge movements. Labeling proved to be insensitive to changes in membrane potential, temperature and pH. Labeling was relatively insensitive to changes in Na^+^ and/or Cl^−^ concentration when Na^+^ was replaced with cations such as choline or tetraethylammonium or when Cl^−^ was replaced with anions such as gluconate or MES. However, substitution of Cl^−^ with valproate significantly increased the rate of labeling with MTSET, driving it to completion and leading to a complete loss of transporter function.

GAT1 contains 14 cysteine residues, of which only three (C74, C164, C173) are predicted to be exposed to the extracellular fluid. C74 is in the first transmembrane domain near the membrane/extracellular fluid interface. C164 and C173 are located in the extracellular loop connecting transmembrane domains 3 and 4, and there is evidence that these as well as homologous residues of the related serotonin and dopamine transporters form a disulfide bridge under most conditions (Chen et al. 1997, 2007). Site-directed mutagenesis revealed that the site of covalent sulfhydryl modification of GAT1 is most likely the cysteine at position 74. Our data cannot rule out the possibility that the functional consequence of labeling is due to modification of a cysteine residue other than C74, whose accessibility is altered in the mutant C74A. However, considering the totality of our data set (insensitivity of C74A to MTSET and virtually identical steady-state state and presteady-state kinetic properties of WT and GAT1 C74A), it is plausible to conclude that C74 is very likely the only functionally sensitive cysteine residue that is labeled by MTSET. Extensive functional characterization suggested that GAT1 C74 is only partially accessible from the extracellular fluid. However, once covalently modified with MTSET, GAT1 is incapable of undergoing the conformational changes necessary for presteady-state transients or Na^+^/Cl^−^/GABA cotranslocation across the plasma membrane.

Our first goal was to optimize the experimental conditions under which all GAT1 copies in the plasma membrane could be rapidly labeled with MTSET, while maintaining cellular and plasma membrane integrity such that accurate biophysical measurements could be carried out. Initial time and concentration dependence of MTSET labeling of GAT1 demonstrated that, under most labeling conditions, very long incubation times at high MTSET concentrations are required to approach complete labeling of all transporter copies in the plasma membrane. This observation is not consistent with the very high intrinsic reactivity of methanethiosulfonate reagents toward free thiol groups (Karlin and Akabas 1998) and suggests that C74 is only partially exposed to the extracellular fluid, such that accessibility of this site to MTSET is restricted (Yu et al. 1998). Indeed, sequence alignments of GAT1 with the related bacterial leucine transporter (LeuT_Aa_) and other members of the SLC6A family of transporters, as well as homology models of GAT1 built using the high-resolution structure of LeuT_Aa_ as a template, strongly suggest that C74 is in the first transmembrane domain near the membrane–extracellular fluid interface and is partially buried in the membrane (Yamashita et al. 2005; Beuming et al. 2006; Livesay et al. 2007). As a practical matter, the relatively short half-life of MTSET in physiological buffers (~10 min) also necessitates the use of experimental conditions that can achieve faster labeling of GAT1.

As GAT1-mediated GABA transport is Na^+^- and Cl^−^-coupled, we investigated the degree of MTSET labeling when Na^+^ or Cl^−^ was isosmotically replaced with various cations/anions in the labeling buffer. It is well established that Na^+^ and/or Cl^−^ binding events to GAT1 cause transporter conformational changes (Mager et al. 1993, 1996; Golovanevsky and Kanner 1999; Lu and Hilgemann 1999b; Li et al. 2000; Bicho and Grewer 2005; Karakossian et al. 2005; Meinild et al. 2009). We reasoned that conformational changes induced by Na^+^ and/or Cl^−^ binding may alter the accessibility of C74 to the extracellular space and that, therefore, it was plausible to examine the degree of labeling in buffers that lacked either one or both of these cosubstrates. For most ion replacement conditions tested, only a modest enhancement was observed in the ability of MTSET to label GAT1, as judged by the reduction in the GABA-evoked current (see Fig. 2). Two notable exceptions were replacement of Na^+^ with Li^+^ and replacement of Cl^−^ with valproate.

Consistent with previous reports on GAT1 and the serotonin transporter (SERT), we observed a substantial reduction in transport activity following MTSET labeling when Li^+^ replaced Na^+^ in the labeling buffer (Figs. 2,  4) (Ni et al. 2001; Meinild et al. 2009). In SERT, the increased accessibility of C109 (homologous with GAT1 C74) is due to Li^+^ binding rather than the absence of Na^+^ (Ni et al. 2001), and this is consistent with our findings in GAT1 (Fig. 2). In GAT1, Li^+^ by itself is not capable of driving Li^+^/Cl^−^/GABA cotransport as the Li^+^-bound transporter conformation is distinct from the Na^+^-bound conformation (Meinild et al. 2009). However, in the presence of low concentrations of Na^+^, Li^+^ can substitute for Na^+^ at one of the cation-binding sites, allowing for Li^+^/Na^+^/Cl^−^/GABA cotransport (MacAulay et al. 2002; Meinild and Forster 2012). Thus, in the presence of Li^+^ (Na^+^ absent) GAT1 assumes a distinct conformation with increased accessibility of C74 to the extracellular fluid.

Remarkably, the most significant environmental condition which favored GAT1 labeling by MTSET was when Cl^−^ was replaced with valproate (Figs. 2,  4). Valproate increases the turnover rate of the GATs via an allosteric mechanism that involves increasing the rate of Na^+^ binding to the transporter, the transition that is thought to be the rate-limiting step in the transport cycle (Whitlow et al. 2003; Soragna et al. 2005). Our data show that in the presence of valproate complete MTSET labeling of GAT1 can be accomplished within 10 min (Fig. 4c) and suggest that any future attempts to utilize C74 to label GAT1 in the plasma membrane should use valproate as the major anion in the labeling buffer.

Interestingly, the presence of GABA protected GAT1 from MTSET labeling (Fig. 2) (Zhou et al. 2004). This suggests that during the normal translocation cycle (i.e., Na^+^/Cl^−^/GABA cotranslocation) there is a reduction in the probability (i.e., time) that C74 is exposed to the extracellular fluid and, hence, accessible to covalent modification by MTSET.

We found it surprising that the accessibility of GAT1 C74 to the extracellular fluid and, hence, MTSET was not altered by changes in experimental conditions such as membrane potential, temperature or pH. GAT1 function is highly voltage-dependent, and indeed, voltage-clamp fluorometric measurements have provided strong evidence for conformational changes evoked by voltage jumps (Li et al. 2000; Meinild et al. 2009). Moreover, the rates of both GAT1 steady-state and presteady-state kinetic properties are very temperature-dependent (Binda et al. 2002; Gonzales et al. 2007). Finally, changes in the extracellular pH have been reported to alter GAT1 function (Grossman and Nelson 2002, 2003). Nonetheless, none of these conditions appeared to play a significant role in the ability of MTSET to label GAT1, suggesting that the global conformational changes caused by these factors do not lead to a change in the accessibility of C74 to the extracellular fluid. When labeling was performed at pH 5.0, there appeared to be a decrease in the efficacy of MTSET; however, this was very likely due to the significantly decreased fraction of GAT1 C74 in the deprotonated thiol form—the form that can nucleophilically attack and, thus, react with MTSET. With a p*K*_a_ of ~8.37, only 0.04 % of C74 thiol groups are predicted to be in the deprotonated form at pH 5.0 (vs. 9.7 and 19.0 % at pH 7.4 and 9.0, respectively). Indeed, there appeared to be a correlation between the fraction of C74 in the deprotonated form and loss of transporter function following MTSET treatment (13, 47 and 56 % at pH 5.0, 7.4 and 9.0, respectively) (see Fig. 3 for raw data).

Having found improved experimental conditions for sulfhydryl modification of GAT1 C74 with MTSET (i.e., labeling in the presence of valproate), we wished to understand the mechanism by which MTSET labeling of GAT1 C74 leads to a reduction in the GABA-evoked current ($I_{\text{NaCl}}^{\text{GABA}}$). In principle, the reduction in $I_{\text{NaCl}}^{\text{GABA}}$ may be due to (1) a change in the transporter ion/GABA flux coupling ratio (i.e., change in ion/substrate transport stoichiometry); (2) a significant increase in the apparent affinity constant (*K*_0.5_) values (i.e., reduction in the apparent affinity) for the transporter cosubstrates Na^+^, Cl^−^ and GABA; (3) a reduction in transporter turnover rate (i.e., the rate at which ion/GABA molecules are cotranslocated across the plasma membrane per unit time); or (4) a complete loss of GAT1 GABA transport activity when C74 is chemically modified by MTSET. To study each of these possibilities, we characterized the current that remained after partial labeling of GAT1 with MTSET. Partial labeling was achieved by incubating GAT1 with 1 mM MTSET for 5 min (at 21 °C, *V*_m_ = −50 mV, pH 7.4) in order to achieve ~50 % reduction in $I_{\text{NaCl}}^{\text{GABA}}$ (see Figs. 1, 2, 3, 4).

The results of uptake under voltage-clamp experiments suggest that the current that remains after MTSET sulfhydryl modification of GAT1 has the same ion/GABA flux coupling ratio as that mediated by untreated GAT1: two net positive charges for every GABA molecule translocated across the plasma membrane (Fig. 5). GAT1 exhibits tight coupling of charge flux and GABA flux under a variety of experimental conditions, which suggests a fixed ion/substrate transport stoichiometry (Gonzales et al. 2007; Matthews et al. 2009). Therefore, if labeling with MTSET alters the transport stoichiometry, the ratio of charge flux to GABA flux should shift from the known value of 2. Our results do not support this possibility. Thus, after MTSET treatment, the GABA-evoked current is mediated by transporters that operate with a normal Na^+^/Cl^−^/GABA coupling stoichiometry.

After MTSET treatment, the remaining GABA-evoked current had the same steady-state and presteady-state kinetic parameters, as well as pharmacological properties, as those of untreated GAT1 (Figs. 6, 7, 8). Thus, a reduction in the apparent affinity of the cosubstrates cannot account for the observed reduction in $I_{\text{NaCl}}^{\text{GABA}}$ after MTSET treatment.

After MTSET treatment, the GATs that contribute to the observed macroscopic signal have the same turnover rate as that of untreated GAT1 (Fig. 9). While the exact turnover rate of the GATs and, in fact, most electrogenic Na^+^-coupled transporters has been elusive due to technical limitations (Gonzales et al. 2007), we have at our disposal a simple measurement that provides an index of the transporter turnover rate. The ratio of the maximum steady-state GABA-evoked current (*I*_max_) to the maximum presteady-state charge moved in response to voltage pulses (*Q*_NaCl_ or *Q*_max_) correlates with the transporter turnover rate (Loo et al. 1993; Mager et al. 1993; Eskandari et al. 1997; Sacher et al. 2002; Whitlow et al. 2003; Karakossian et al. 2005; Gonzales et al. 2007). These two parameters are independently measured in individual GAT1-expressing cells, and each is directly related to the total number of functional transporters in the plasma membrane (Zampighi et al. 1995; Eskandari et al. 1997, 1998; Gonzales et al. 2007). Our results demonstrated that, while both *I*_max_ and *Q*_max_ are reduced after labeling (see Figs. 1 and 8), the ratio of *I*_max_ to *Q*_max_ was not altered following MTSET labeling (Fig. 9), suggesting that both parameters were reduced proportionally and that the transporter population that gives rise to these measurements operates with the same turnover rate.

All in all, our data suggest that the observed current following partial labeling with MTSET is mediated by an unlabeled transporter population that operates normally. Transmembrane domain 1 is thought to play a prominent role in GAT1 function by lining the permeation pathway and containing determinants for substrate specificity (Mager et al. 1996; Zhou et al. 2004; Yamashita et al. 2005). It is highly unlikely that the bulky, positively charged MTSET, covalently bonded to C74 at the extracellular end of transmembrane domain 1, does not alter the transport stoichiometry or any of the relevant steady-state, presteady-state or inhibition kinetic parameters of GAT1. It is more likely that the observed GABA-evoked current following MTSET exposure is mediated by transporters that were simply never labeled. We conclude that MTSET labels GAT1 at C74 and that such covalent sulfhydryl modification renders that transporter completely nonfunctional and electrically silent.

At this time, it is difficult to reconcile our data with those reported previously using the voltage-clamp fluorometry method (Li et al. 2000; Meinild et al. 2009). The totality of our data suggests that labeling C74 with MTSET renders the transporter nonfunctional as all known electrical signals of GAT1 are abolished after labeling. These signals include the GABA-evoked steady-state current as well as voltage-evoked presteady-state charge movements. Thus, at first glance, it appears that a fluorophore-conjugated C74 is not a suitable experimental model by which to examine transporter conformational changes that are relevant to those operating during the normal transport cycle. However, previous measurements obtained with the voltage-clamp fluorometry method suggest that GAT1 conjugated (reportedly at C74) with TMR6M exhibits voltage-induced changes in fluorescence intensity and that the time course of these transitions corresponds well to the relaxation time constants of the presteady-state charge movements. Our data show that TMR6M is capable of labeling both WT GAT1 and the mutant GAT1 C74A (see Fig. 1d), suggesting that, in addition to C74, this agent labels other cysteine residues, possibly those buried in the membrane or even exposed to the cytoplasmic space. Clearly, additional studies are needed to better understand the electrophysiological and fluorescence signals following GAT1 labeling with TMR6M. A challenge with selecting suitable fluorescent probes is that their lipid solubility coefficients are generally not known or cannot be estimated (for proprietary molecules). We propose that the simple assay shown here on GAT1 C74A may be performed in order to assess the ability of the fluorophore to cross the plasma membrane and, hence, gain access to internally exposed cysteine residues.

A sequence alignment for members of the neurotransmitter/Na^+^ symporter family (NSS) reveals that the cysteine at position 74 of GAT1 is conserved in most of the mammalian members of the family. However, each family member is predicted to have different numbers of extracellularly oriented cysteine residues, making it difficult to utilize a single residue for labeling purposes without engineering cysteine mutants against a cysteine-less background. Moreover, very different observations have been reported regarding the functional consequence of labeling with the membrane-impermeant MTSET. For example, WT dopamine and norepinephrine transporters appear to be insensitive to functional modification by extracellularly applied MTSET (Sucic and Bryan-Lluka 2005). The SERT exhibits significant functional sensitivity to MTSET only when Li^+^ is the major extracellular cation, and this effect is abolished in the C109A mutant, where SERT C109 is homologous to GAT1 C74 (Chen et al. 1997; Ni et al. 2001; Sucic and Bryan-Lluka 2005). In the glycine transporter (GlyT1b), MTSET leads to a reduction in both steady-state and voltage-evoked presteady-state currents, and this effect is eliminated in the corresponding cysteine-to-alanine mutant (Roux et al. 2001). The basic observations presented here on sulfhydryl modification of GAT1 with MTSET are similar to those reported for the glycine transporter GlyT1b (Roux et al. 2001).
